# Variations of Phosphorous Accessibility Causing Changes in Microbiome Functions in the Gastrointestinal Tract of Chickens

**DOI:** 10.1371/journal.pone.0164735

**Published:** 2016-10-19

**Authors:** Bruno Tilocca, Maren Witzig, Markus Rodehutscord, Jana Seifert

**Affiliations:** Institute of Animal Science, University of Hohenheim, Stuttgart, Germany; Max Rubner-Institut, GERMANY

## Abstract

The chicken gastrointestinal tract (GIT) harbours a complex microbial community, involved in several physiological processes such as host immunomodulation and feed digestion. For the first time, the present study analysed dietary effects on the protein inventory of the microbiome in crop and ceca of broilers. We performed quantitative label-free metaproteomics by using 1-D-gel electrophoresis coupled with LC-MS/MS to identify the structural and functional changes triggered by diets supplied with varying amount of mineral phosphorous (P) and microbial phytase (MP). Phylogenetic assessment based on label-free quantification (LFQ) values of the proteins identified *Lactobacillaceae* as the major family in the crop section regardless of the diet, whereas proteins belonging to the family *Veillonellaceae* increased with the P supplementation. Within the ceca section, proteins of *Bacteroidaceae* were more abundant in the P-supplied diets, whereas proteins of *Eubacteriaceae* decreased with the P-addition. Proteins of the *Ruminococcaceae* increased with the amount of MP while proteins of *Lactobacillaceae* were more abundant in the MP-lacking diets. Classification of the identified proteins indicated a thriving microbial community in the case of P and MP supplementation, and stressed microbial community when no P and MP were supplied. Data are available via ProteomeXchange with identifier PXD003805.

## 1. Introduction

The chicken gastrointestinal tract (GIT) microbiome comprises more than 900 species of microorganisms [1]. This diverse microbiome establishes a complex network of interactions with the host playing an important role for the animal growth and health, since it is involved in several physiological processes such as modulation of the host immune system as well as breakdown and digestion of the feedstuff [1, 2]. Microbial composition changes longitudinally and radially along the gastrointestinal tract, since each intestinal section has its own characteristic “microenvironment” suitable for a better colonization by specific microbial species [2, 3]. In addition, changes of the phylogenetic structure are also caused by animal genetics, dietary composition and other environmental factors [2, 4].

To date, several studies investigated the chicken microbiota based on cultivation and 16S rRNA gene analysis (for reviews see [2, 4–6]). The potential microbial functions were described in only a handful of studies using shotgun sequencing to analyze the metagenome in either cecal or fecal samples from chickens [7–10]. In a recent study, Sergeant *et al*. [9] analyzed the metagenome of the cecal content from a single bird in order to assess the bacterial phylogenetic distribution and its potential activity. Another recent study investigated the microbiome of two pooled fecal samples based on 16S rRNA gene sequencing and metaproteomics in order to understand the metabolic processes in the gut of a healthy chicken [11]. Polansky et al. investigated the cecal microbiome of chickens at different ages, including 1-week-old chickens after inoculation with cecal extracts from hens of different ages, on the attempt to explain colonization patterns and predict the most promising probiotic genera for cecal colonization of newly hatched chickens [12]. Despite the extensive and heterogeneous ensemble of published studies, in-depth investigations about the possible functional changes of the microbiome of the chicken gut challenged with different dietary treatments are missing.

Phosphorous (P) is an essential macro element involved in a multitude of physiological processes such as bone development, growth and productivity of livestock. In plant seeds, organic phosphorous is mainly present as phytate, the salt of *myo*-inositol 1,2,3,4,5,6-hexakis (dihydrogen phosphate) (phytic acid; InsP_6_). InsP_6_ is only partially digestible for many monogastric animals such as chicken [13, 14]. Here, P bioavailability can be improved by phytases, phosphatases responsible for phytic acid dephosphorylation and release of P, available for the animal absorption in the intestine. Since endogenous phytases in chicken are less efficient and their activity depends on dietary phosphorous and cholecalciferol [15, 16], standard chicken diet formulation requires supplementation of mineral P and microbial phytase (MP) in order to reduce the problem of low endogenous phytase activity and ensure adequate P provision to the animal [17]. Additional phytase dosage also helps to reduce the need for supplementation of mineral P in the feed formulation. This leads to a consequent reduction of P excretion which has a great significance for the ecological problem of water eutrophication and saving of P, a limited resource of global importance. Feeding diets with mineral levels under or above the optimal requirement may trigger alterations in microbial activity and composition, on the attempt to fulfil the nutritional requirements or by the alteration of the physico-chemical environment in the gut lumen [17–19].

To the best of our knowledge, no studies are published so far investigating the functional changes of the bacterial community inhabiting different GIT sections and correlating it to the different effects of broiler diets either supplemented with mineral P and/or MP. Witzig and colleagues (2015) characterized changes in bacterial phylogenetic compositions along the GIT of broilers fed with different mineral P and MP supplemented diets by T-RFLP and 16S rRNA gene sequencing [20]. They showed an effect of mineral P and MP towards the abundance of certain *Lactobacillus* spp. especially in the crop and a possible influence of MP in the cecal community. Here, we used this feeding experiment to analyze proteins extracted from crop and cecal bacterial communities to obtain indications for possible functional changes in the microbiome of these gut sections. A label-free quantitative (LFQ) metaproteomic approach was used for the assessment of the protein phylogenetic composition and abundance. Discussion on the detected functional pathways and the change of abundance of certain proteins in the diverse dietary treatments is given.

## 2. Materials and Methods

### 2.1 Animal experiment

Animal handling and treatments of the present study were approved by the animal welfare commissioner of the University of Hohenheim (internal experiment number T98/12 TE) in accordance with the German welfare regulations (documents are included in the supplementary information as S1 File). A master of poultry farming did the animal experiment and euthanasia as regulated by the German law.

Samples were obtained from an animal experiment described in detail by Zeller et al. [14]. A schematic overview of the experimental workflow is provided in Figure A in S2 File. A total of 1,140 unsexed broiler hatchlings (Ross 308) were obtained from a local hatchery (Brüterei Süd GmbH and Company KG, Regenstauf, Germany). One hundred and eighty birds were housed in 18 pens (10 animals each) to investigate tibia mineralization, details on [14]. The remaining 960 animals were housed in 48 pens (20 animals each). On day 15 of age, broiler chickens were assigned to six experimental treatments (8 pens/treatment) until day 25 of age. On day 25, birds were sacrificed by carbon dioxide asphyxiation following anesthesia in a gas mixture (35% CO_2_, 35% N_2_, and 30% O_2_). From six pens per treatment, four animal each, were chosen for DNA-based investigation of the chicken GIT microbiota as detailed in [20]. Out of these, 4 animals each from two pens per treatment (48 animals in total) were chosen for the metaproteomic investigations. Remaining animals were used for investigation on inositol phosphate degradation [14]. The contents of crop as well as of cecal sections were separately collected and homogenized on a pen basis, yielding two pooled cecal and crop samples, respectively, per dietary treatment, for a total of twenty-four samples (Figure A in S2 File).

### 2.2 Experimental diets

All animals were fed a commercial broiler starter diet until day 14 of age. At day 15 the experimental diets were fed, all consisting of a basal diet [21] containing adequate levels of all nutrients, according to the recommendations of the German Society for Nutritional Physiology (GfE), with the exception of mineral P and calcium. Three diets contained P exclusively deriving from plant sources (BD–), whereas in another three diets additional P was supplied as mono-calcium phosphate (BD+). Diets of BD—and BD+ groups were further supplemented with 0, 500 and 12500 U/kg respectively of an *Escherichia coli* microbial phytase product (Quantum Blue, EC 3.1.3.26, supplied by AB Vista, Marlborough, UK), allowing a further distinction between MP0 and MP+ (MP500 and MP12500) diets.

Insights on the manufacturing of the diets as well as details on the diets composition and analysis performed on the experimental diets, are provided in the S1 Table and in reference [14].

### 2.3 Sample preparation

Twenty four pooled samples were kept on dry ice during their transport to the laboratory and stored at -80°C until their analysis. After thawing at 4°C, bacterial cells were separated by using a previously described method [22] with modifications. Briefly, aliquots of 0.5 g of pooled samples were resuspended by vortexing in 15 mL washing buffer (50 mM Na_2_HPO_4_, 0.1% Tween 80, [pH 8.0]). Samples were then incubated for 10 min in a sonication bath (amplitude 50%, 0.5 cycle), shaken for 20 min in a reciprocal shaker at 100 oscillations/min and centrifuged at low speed (200 x g, for 15 min, 4°C). Supernatant containing the bacterial cells was collected in a 50 mL tube and the remaining pellet was subjected to further 4 rounds of the whole protocol, for a total of five rounds. Bacterial cells in the pooled supernatant were pelleted by centrifugation at 15,000 x g for 30 min at 4°C and subjected to protein extraction.

### 2.4 Protein extraction, quantitation, digestion

Recovered cells were resuspended by vortexing with 100 μL extraction buffer (2% SDS, 20 mM Tris-HCl [pH 7.5]) and mixing at 1400 rpm for 10 min at 60°C. Each sample was then mixed with 1 mL Tris-HCl buffer (20 mM Tris-HCl [pH7.5], 0.1 mg/mL MgCl_2_, 1 mM phenylmethanesulfonyl fluoride, 1 μL/mL benzonase (Novagen, 99% 25 U/ μL). Cell lysis was ensured by 5 rounds of 1 min ultra-sonication using a sonication probe (amplitude 50%, cycle 0.5) and 1 min rest on ice. After 10 min shaking at 1400 rpm, 37°C, samples were centrifuged at 10,000 x g for 15 min at 4°C. Extracted proteins contained in the supernatant were quantified with the Quick Start^™^ Bradford protein assay (Bio-Rad, Hercules, USA) following the manufacturer’s instructions. Approximately 50 μg of the extracted proteins were precipitated by incubation (30 min at 4°C) with precooled 20% trichloroacetic acid (TCA). Protein pellet was resuspended in 25 μL Laemmli-buffer for 5 min at 95°C before being purified on a one-dimensional sodium dodecyl sulphate-polyacrylamide gel electrophoresis (SDS-PAGE, 4% stacking gel, 20 mA; 12% running gel, 40 mA). Proteins were trapped in the first centimeter of the separation gel and an overnight in-gel digestion using in-gel trypsin (Promega) was done on the complete part of the protein-loaded gel piece [23, 24]. Recovered peptides were purified and desalted by using Zip-Tip C18 tips (Millipore, Billerica, USA), dried at the SpeedVac and resuspended in 5% acetonitrile (ACN) / 0.1% trifluoroacetic acid (TFA) before the LC-MS/MS measurement.

### 2.5 LC-MS/MS analysis

Five microliters of the resuspended peptide mixture were measured in three technical replicates by using Q-Exactive Plus mass spectrometer (Thermo Fisher Scientific, Darmstadt, Germany) faced with EASY-nLC 1000 (Thermo Fisher Scientific) equipped with an EASY-Spray PEPmap column (50 cm x 75 μm inner diameter) packed with C18 resin, 2 μm particles, 100 Å pore size (Thermo Fisher Scientific). Peptides were loaded onto the HPLC column through solvent A (0.1% formic acid) at a flow rate of 500 nl/min and eluted over a solvent B (80% ACN in 0.1% formic acid) gradient ranging from 5% to 35% in the first 200 min and from 35% to 45% in the following 40 min.

MS/MS instrument was set to positive ion mode. Full scan was acquired in the mass range from m/z 300 to 1650 in the Orbitrap mass analyzer at a resolution of 120,000 followed by HCD fragmentation of the 12 most intense precursor ions. High resolution MS/MS spectra were acquired with a resolution of 30,000. The target values were 3*10^6^ charges for the MS scans and 1*10^5^ charges for the MS/MS scans with a maximum fill time of 25 ms and 45 ms, respectively. Fragmented masses were excluded for 30 s after MS/MS. Spectra de-noising was performed prior to peptide identification by considering the only top 12 peaks in a window of 100 Da width.

### 2.6 Bioinformatic data analysis

To reduce the false discovery rate of peptide identification and for a better evaluation of protein abundance a two-step approach for bioinformatics data analysis was chosen. Briefly, proteins identified in the first database-dependent search are used to build a second sample-specific database, used for the second database-dependent search. The smaller size of the latter database enabled for a high coverage of the metaproteome as well as a reduced number of false positive inference [25, 26].

Acquired raw data were at first processed using Thermo Proteome Discoverer software (v.1.4.1.14), Mascot (v. 2.4) and searched against NCBI-nr bacteria and chicken databases (release July 12th, 2014) in order to export a protein fasta database. Methionine oxidation was set as variable modification and carbamidomethylation of cysteine as fixed modification. Default settings of the software were kept, these include protein grouping with peptide confidence set on “high” and delta Cn of 0.1. The Percolator node supporting a strict maximum parsimony principle was activated with a false discovery rate of 1%. In the second process, exported protein fasta file from the first search was subsequently used as *in-house* database for the peaks alignment and mass re-calibration in the first step of the MaxQuant search. MaxQuant software (v.1.5.1.2) set on LFQ modality was used for peptide identification and protein IDs inference. In the second step of MaxQuant analysis, raw data were independently searched against UniProtKB databases (release October 2014) bacteria (UniProt ID 2, 18976242 entries) and chicken (UniProt ID 9031, 82439 entries). In the database search, cysteine carbamidomethylation was set as fixed modification and methionine oxidation as variable modification. Two missed cleavage sites were allowed for protease digestion and peptides had to be fully tryptic. All other parameters of the software were set as default, including a peptide and protein FDR < 1%, at least 1 peptide per protein, precursor mass tolerance of 4.5 ppm after mass recalibration and a fragment ion mass tolerance of 20 ppm.

The mass spectrometry proteomics data are publicly available in the ProteomeXchange Consortium via the PRIDE [27] partner repository with the dataset identifier PXD003805.

Further insights on the identified peptides and their implication for protein IDs inference, are provided in the S2 and S3 Tables.

Phylogenetic information were inferred on basis of the protein description outputted from the MaxQuant searches. These, in turn, are gathered from the protein annotation of the chosen database (*i*.*e*. UniProt KB).

LFQ abundances from MaxQuant´s output results were subjected to statistical analysis by using Primer6 v.6 statistical software (PRIMER-E, Plymouth, UK). Principal coordinate analysis (PCoA) was calculated on the basis of the Bray-Curtis similarity matrix [28].

Statistical difference between diet treatments was calculated by performing analysis of variance with permutations (PERMANOVA). Statistically different treatments were then subjected to SIMPER analysis in order to isolate proteins responsible for dissimilarity between pairs of groups with a cut-off threshold of 99.99% [29]. Selected proteins were functionally classified into COG and KEGG categories by using WebMGA on-line tool [30] with an e-value cutoff of 10^−3^ and exclusively considering the best hit.

Cladograms visualizing the dietary effects on the structure of the chicken´s crop and cecal microbiome were drawn using the Galaxy on-line tool. It implements the computation of the Linear Discriminant Analysis [21] between the technical triplicates of each experimental diet group (*n* = 2). Here, the Kruskal-Wallis test is performed to check whether differences between the experimental diets are statistically significant (p <0.05). Only bacterial families showing discriminative effects with respect to the diets were considered and ranked according to the effect size with which they differentiate the diets [31].

Heat-Maps for phylogenetic composition across the different diet treatments and functional classification of the identified proteins were drawn using heatmap.2 provided by the gplots package [32] implemented in R v.3.1.2 software (http://www.R-project.org).

## 3. Results and Discussion

### 3.1 Chicken proteome

Despite using a protocol to analyze microbial proteins, it is a common phenomenon that eukaryotic proteins are always co-extracted and processed during the metaproteomics workflow [33–35]. In this study, a total of 248 and 405 host proteins were identified in the crop and cecal section, respectively, which were investigated in order to highlight the possible presence of specific transporters or any other activity related to the adaptation to the changing dietary conditions. A complete list of the identified host proteins is given in S4 Table. Functional classification of the identified proteins was performed by categorization of their abundance intensity into COG classes and KEGG pathways. In addition, subcellular classification [36] of the chicken proteins reveals that only a small fraction of the proteomes were plasma membrane proteins (6.9 and 14.8% on average for crop and ceca respectively, data not shown). No statistical differences in chicken protein abundances were observed between the diets in both GIT sections. The reason may be found in the relatively low protein numbers, which were obtained by the sample preparation protocol, which favored prokaryotic cells [22].

### 3.2 Analysis of the bacterial metaproteome

The used sample preparation protocols yielded a total of 381 and 1,719 bacterial proteins for crop and ceca sections respectively, with 3.1% of the total proteins shared among both sections. The relatively low identification rate in the crop section is probably explained by the low bacterial abundance typical of this section and the high amount of feed residues, which were co-extracted [37]. Details on the number of identified proteins and peptides in each sample, as well as a general overview of the abundance intensities of proteins in both GIT sections along with their grouping into KEGG biochemical pathways, are summarized in Figure B in S2 File and S5 Table.

**Fig 1:**
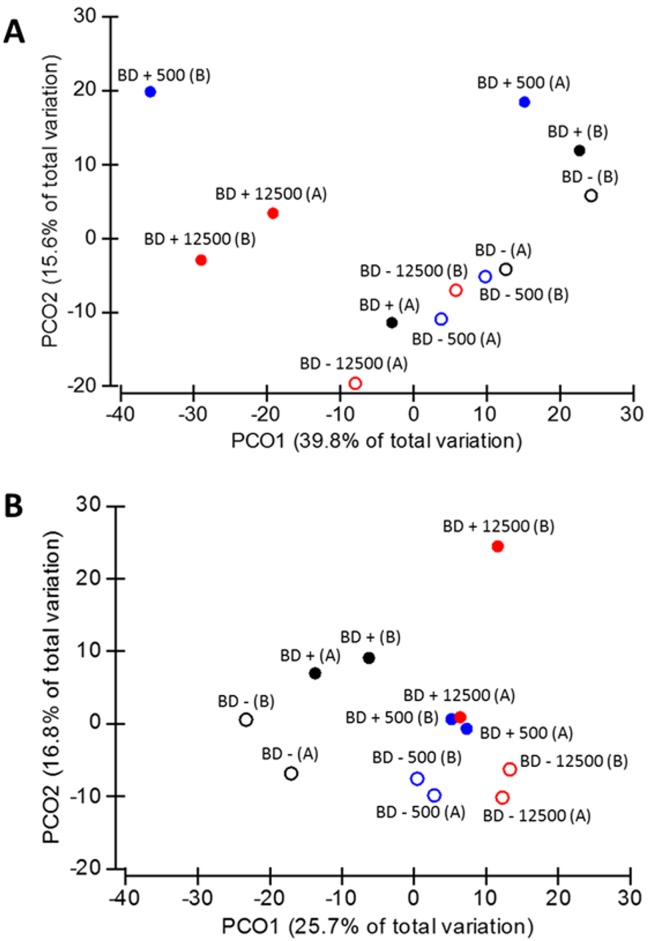
Principal coordinate analysis of the crop (A) and ceca (B) microbiome at different dietary treatments. Open shapes refer to diet without P supplementation, full shapes concern diets with P addition. Black, blue and red colors refer to MP0, MP500, and MP12500, respectively.

Out of the overall dataset, PCoA analysis was performed to ordinate the samples depending on the different dietary treatments (Fig 1). In the crop section, 38.9% of the total variation was observed in the PCoA1, where metaproteomes of the samples without mineral P supplementation clustered together and drift apart from the samples supplemented with mineral P (*p* = 0.043, Fig 1A). PCoA analysis of the cecal samples showed a clustering of the microbial proteins from birds fed with mineral P- supplied diets and a separation from treatments without mineral P supplementation, which was not significant (*p* > 0.05, Fig 1B). A very clear distinction was shown between the MP-containing diets that clustered apart from the MP-lacking diets (*p* = 0.008).

Experimental error due to the possible variability of the analytical techniques was controlled by averaging the technical triplicates measured for each sample. The PCoA analysis of cecal samples (Fig 1B) also showed that the pairs of biological duplicates were close together, with an average similarity of 72.2% (ranging from 69.0% to 84.5%), meaning that observed differences represent a “true” biological difference induced by our experimental treatments.

### 3.3 Bacterial taxonomy of the proteins

**Fig 2:**
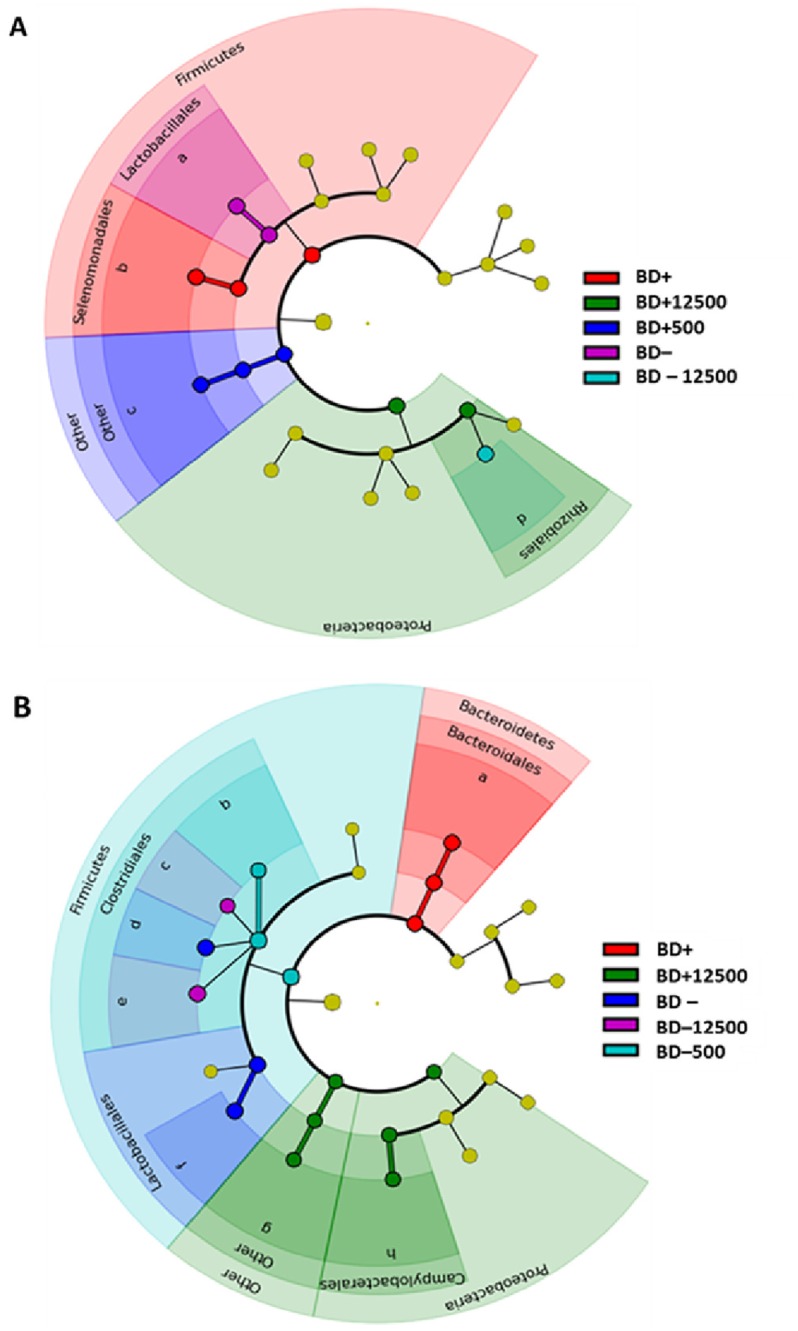
Dietary effect on phylogenetic composition of the chicken´s crop (A) and cecal (B) microbiome. Cladograms of both sections show a comparative evaluation of the experimental treatments effects on the structure of the chicken´s GIT microbiome. Effects are calculated through LDA Effect Size [53], a two-module algorithm. In the first module, technical triplicates of each dietary group (*n* = 2) were subjected to non-parametric Kruskal-Wallis test to detect features with significant differential abundance with respect to the experimental treatments. In the second module, tabular abundance data formatted in the previous module are subjected to Linear Discriminant Analysis [21] to estimate the effect size of each differentially abundant feature. The only diets and bacterial families showing statistical significance (p<0.05) in the previous statistical tests are visualized in the figures. Yellow dots refer to bacterial specimens whose protein pattern and abundance did not score a statistical significant effect (p>0.05) in any of the experimental diets. Bacterial families legend: **(A)** a: *Lactobacillaceae*; b: *Veillonellaceae*; c: *Other families*; d: *Bradyrhizobiaceae*. **(B)** a: *Bacteroidaceae*; b: *Clostridiaceae*; c: *Eubacteriaceae*; d: *Lachnospiraceae*; e: *Ruminococcaceae*; f: *Lactobacillaceae*; g: *Other families*; h: *Helicobacteraceae*.

The phylogenetic composition of the bacterial proteins extracted from crop and ceca was determined based on the abundance values of the proteins belonging to each bacterial family. These values were summarized and only families with a cumulative abundance greater than 3% or 1% of the total were considered for phylogenetic analysis of crop and ceca samples, respectively (Fig 2, Figure C in S2 File). The abundance of each bacterial family in respect to the experimental diets is shown for the crop (Fig 2A) and cecal (Fig 2B) samples. Only bacterial families showing a discriminative effect (*p* < 0.05) with respect to the diets were considered and ranked according to their contribution in the different experimental treatments. This rank is graphically displayed as width of the cladogram portion attributed to each bacterial family. In addition, a phylogenetic assessment was done considering only families expressing at least one phylogenetic marker protein (*i*.*e*. highly conserved proteins employed as marker for phylogenetic analysis) [38–41] (S6 Table). This assessment reflected qualitatively the phylogenetic distribution of the total proteins.

In general, phylogenetic distribution of the crop metaproteomes showed a reduced bacterial diversity and a high inter-individual diversity among all dietary treatments causing the unpaired scattering of the biological duplicates in the PCoA analysis (Fig 1A). In accordance with other studies on the chicken´s GIT microbiota composition (for reviews see [2, 4, 6]), proteins belonging to *Lactobacillaceae* were the most abundant regardless of the diets (Figure C in S2 File). The number of proteins belonging to *Veillonellaceae* increased on average in BD+ diets (36%) when compared with the BD—diets (23%; Figure C panel A in S2 File, Fig 2A). *Veillonellaceae* has been often associated to fiber digestion and short chain fatty acid production [42, 43], therefore its increase in P-supplied diets suggests a potential beneficial effect for the animal growth and performance [44]. With the exception of *Veillonellaceae* and a few other families commonly found in the chicken´s upper GIT (*Propionibacteriaceae*, *Erysipelotrichaceae*, *Eubacteriaceae*, *Clostridiaceae*) [45], other minor bacterial families identified in this study such as *Nocardiaceae*, *Gordoniaceae*, *Bradyrhizobiaceae*, *Rhizobiaceae*, *Moraxellaceae*, *Desulfovibrionaceae* and *Pseudomonadaceae* are more likely to be found in environmental samples [46, 47]. However, all these families were also found in the gut microbiome of humans or other animals [45, 48–50], therefore their presence in the crop microbiome may be either attributed to an intake from the environment (e.g. with the feed) or such bacterial families can be considered as common members of the crop´s microbial fraction. The DNA-based phylogenetic analysis of the same crop samples analyzed by Witzig et al. resembled also the predominance of *Lactobacillaceae* and a decrease of them concomitant to MP supplementation [20]. Other minor bacterial families were either not detected in the DNA-based study or in the present one.

Phylogenetic distribution of the cecal microbial community (Fig 2B) showed a higher phylogenetic diversity of the identified proteins in all dietary treatments and a change in the composition depending on the diets. Proteins belonging to *Bacteroidaceae* showed an average abundance of 14.9% in mineral P-supplied diets regardless of the presence of MP compared to 8.4% abundance in the samples from the diets without mineral P supplementation. Conversely, proteins belonging to *Eubacteriaceae* were more abundant in all diets without mineral P supplementation (4.6%) than with mineral P supplementation (3.9%). Proteins belonging to *Ruminococcaceae* were more abundant in the diets with increasing amounts of MP (25.4% in the MP+ diets *vs*. 11.5% in the MP0 diets) whereas proteins of *Lactobacillaceae* showed a contrary abundance (5.2% in MP+ *vs*. 27.8% in MP0 diets; Fig 2B, Figure C panel B in S2 File). The increased abundance of *Bacteroidaceae* in the BD+ diets as well as the decrease of *Lactobacillaceae* due to the MP supplementation is in line with the results of the DNA-based analysis performed by Witzig et al. [20]. However, no increased abundance of *Bacteroidaceae* due to the dietary MP addition was observed and no *Erysipelotrichaceae* members were found in the present metaproteomic investigation. Nevertheless, a higher bacterial diversity in the microbiome of crop and ceca sections was highlighted with the present approach. The discrepancy in the results from the same samples is imputable to the different methods. The amplification steps of T-RFLP and amplicon pyrosequencing analyses, including a possible primer bias and the presumed overestimation of taxa with a higher number of 16S rRNA genes, are probably the reason of the reduced heterogeneity found in the microbiota composition. On the other hand, this step allows a higher sensitivity to target minor bacterial families which are missing in the metaproteomic approach due to the lack of genomic sequences. Besides these technical issues, the greater number of changes in the bacterial composition highlighted in the present work may be due to the point that a change of abundance of the expressed proteins is detected earlier than change in the number of DNA copies. Consistently, other studies observed this phenomenon. Haange et.al described a higher number of phyla and classes in their metaproteomic dataset than in 16S rDNA sequencing data, while investigating colon mucosa and fecal rats microbiota [34]. Similarly, inconsistencies between DNA- and protein-based microbiota assessment was described by Tang et al. [11]. They also showed that the correlation between the potential and active bacterial community is not always possible since the species identification in proteomics was different to that of 16S rRNA gene sequencing. Contrariwise, comparable results were observed by Polansky and colleagues while considering the cecal microbiota composition as determined by 16S rRNA gene sequencing and through protein mass spectrometry [12].

### 3.4 Abundance of metabolic functions varying between the dietary treatments

A general overview of the global chicken crop and cecal metaproteomes and abundance intensities of the identified proteins grouped into KEGG biochemical pathways and COG categories is shown in the Figure B and D in S2 File, respectively. PERMANOVA analysis of the total datasets of both sections was done to check for significant differences between diets. Crop samples showed that the only pair of treatments MP0 –MP12500 across P factor were statistically significant (*p* = 0.048). Within ceca section, statistical significance was shown by the experimental treatments with and without mineral P supplementation across the MP factor (BD–/BD+; *p* = 0.037), whereas among the MP-containing diets, the pairs MP0/MP500 and MP0/MP12500 showed statistical significance (*p* = 0.025 and *p* = 0.031, respectively).

**Fig 3:**
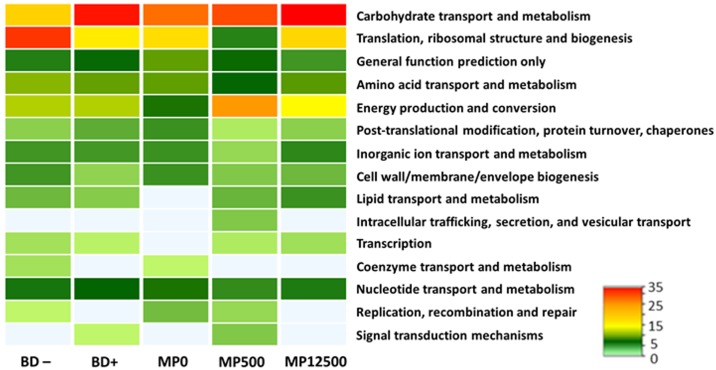
Functional classification of cecal proteins into COG categories. Heat-Map is drawn on the basis of the relative percentages of the proteins of each statistically different treatment. COG classification of crop samples proteins is available in Figure D in S2 File.

The entries of the statistically different treatments were subjected to SIMPER analysis, using a strict cut-off threshold (99.99%), to identify single proteins that caused the dissimilarity between treatment groups. These proteins were functionally classified by grouping them into COG categories (Fig 3). Concerning crop section, the comparison between MP0 and MP12500 treatments showed that the MP0 metaproteome include some unique COG categories expressed at a low relative percentage (Figure E in S2 File). In MP12500 metaproteome, lipid metabolism (I) was uniquely identified and three categories were more abundant in comparison with MP0 such as translation, ribosomal structure and biogenesis (J), carbohydrate transport and metabolism (G) and amino acid transport and metabolism (E) (Figure E in S2 File).

Protein data of the cecal samples showed that proteins belonging to translation, ribosomal structure and biogenesis (J) were more abundant (23%) in the BD- samples than in the BD+ ones (14%) (Fig 3). In contrast, proteins of the carbohydrate transport and metabolism (G) group were more abundant, increasing from 16% in the BD—to 24% in the BD+ diets. Among the MP-containing treatments, some COG categories were found to increase with the addition of MP such as carbohydrate transport and metabolism (G) and energy production and conversion (C); while the lipid transport and metabolism (I) category was solely identified in the MP-containing diets. The post-translational modification, protein turnover, chaperones (O), by contrast, was found to decrease with the MP-addition. This last, together with the increasing category carbohydrate transport and metabolism (G) and lipid transport and metabolism (I) are in agreement with the results obtained in the crop section (Fig 3), suggesting that these changes are particularly triggered by the MP addition. Based on these results, our initial hypotheses that the diets will affect the functional profile of the bacterial communities was accepted. Specifically, mineral P-available diets (BD+; MP+) stimulate a “productive bacterial community” where bacterial resources are focused on complex anabolic functions; while the microbial community present at low mineral P diets (BD–; MP0) is concerned in the stress response mechanisms, suggesting that the mineral P limitation affecting the host health status is also reflected as stress factor for the gut microbiota.

This idea seems to be in accordance with a study of Tang et al., which correlated the expression of proteins involved in metabolic processes of carbohydrate, alcohol and proteins to a thriving microbial community [11]. Protein folding has been linked to the microbial stress response to the high temperature of the chicken body. In our results, the thriving condition is maintained by the adequate nutrients supplementation, while the main stress factor is represented by the lack of P. The great expression of proteins involved in carbohydrate metabolic processes (BD+, MP+) is also supported by another work providing the genetic evidence of numerous enzymes involved in polysaccharide degradation and sugar transport and utilization [9]. Qu et al. observed an enrichment in the “carbohydrate metabolism” SEED subsystem in the cecal microbiota of the control bird when compared to the metabolic potential of a chicken challenged with *Campylobacter jejuni* infection [8]. The metabolic potential of the chicken´s fecal microbiota as assessed by Singh et al. [51] shows that the abundance of the “carbohydrate metabolism” SEED subsystem was stable between the metagenomes of low and high feed conversion rate chickens whereas SEED subsystems related to sulphur metabolism and motility/chemotaxis were statistically different.

### 3.5 Single proteins and pathways highlighting the functional differences

The abundance intensities of the proteins, responsible for significant dissimilarities between experimental treatments in the two GIT sections, were additionally grouped according to KEGG biochemical pathways (Figure F in S2 File). In both sections, major biochemical pathways were identified in all experimental treatments, but only a low number of proteins within these pathways was shared between the different treatments, suggesting a diverse overall activity of the microbial communities depending on the fed diets (Figure F in S2 File, S7 Table). Additionally, differences in the abundance level of the common proteins across the diets were observed, indicating a probable modulation of the highly conserved functions of the microbiota on the attempt to shape an adequate response to the changing environment. Specifically, the higher abundance of the KEGG Orthologous system [52] for “ribosome” (KO 03010) and “aminoacyl-t-RNA biosynthesis” (KO 00970) observed in crop MP12500 samples, as well as the higher abundance of “ABC transporters” (KO 02010) registered in crop MP0 samples, suggest an increased metabolic activity, and an overall effort of the bacterial community to maximize the P uptake respectively for the MP+ and MP0 samples (Figure F in S2 File). Similar trend was observed in the cecal samples. The high-expression of several KOs such as “glycolysis/gluconeogenesis” (KO 00010, BD+ samples) and “starch and sucrose metabolism” (KO 00500, MP+ samples) indicate a productive bacterial community and a coexistence of the bacterial members in a thriving microenvironment. Microbial communities of BD- and MP0 samples by contrast, are more focused on diverse degradation pathways and “ABC transporters”, suggesting harder survival conditions for the bacterial members that must effort on the attempt to maintain the level of required phosphorous above the least threshold (Figure F in S2 File).

**Fig 4:**
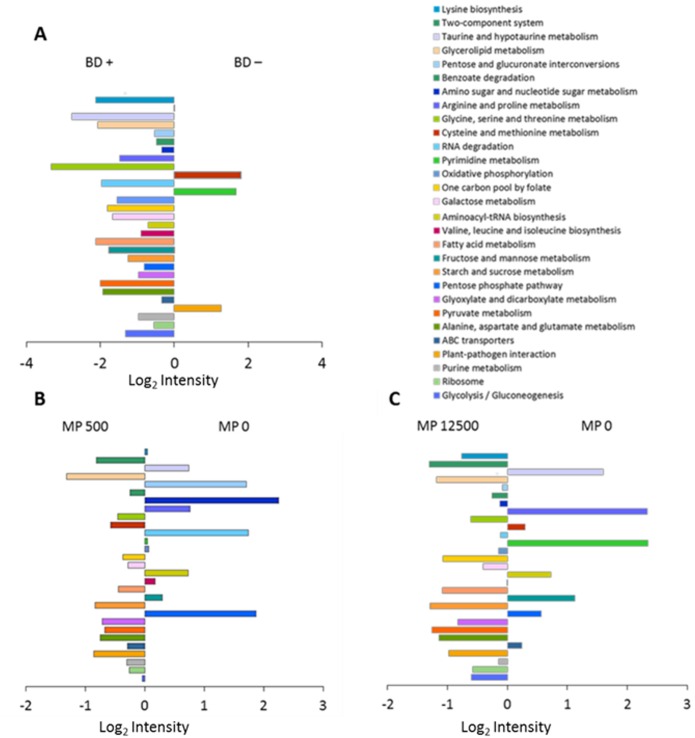
Relevant biochemical pathways between experimental treatments. Comparison of different dietary treatments: **(A)** BD+ and BD–. **(B)** MP500 and MP0. **(C)** MP12500 and MP0 based on the log2 of the ratios between the cumulative intensities of the statistically significant pairs of KOs. Each of the graph´s bars represent a KEGG biochemical pathway. Only pathways with a cumulative abundance greater than 1% of the total are considered in the graph.

A comparative evaluation on how the abundances of the KOs [52] vary in the different diets is shown in Fig 4. In the proteins of the ceca, the majority of pathways are overrepresented in the BD+ diets (Fig 4A). Several KOs were found to be overrepresented in the MP0 treatments such as ‘pyrimidine metabolism’ and ‘arginine and proline metabolism’ (KO 00330). Their abundances decreased with the addition of MP, resulting in a more favorable ratio for the MP0 treatment (Fig 4 panel B and C). Conversely, ‘lysine biosynthesis’ as well as ‘oxidative phosphorylation’ registered an overrepresentation in MP0 in the only pair MP0/MP500, while a further MP addition (MP0/MP12500) led to a slight overrepresentation in the MP12500 sector.

Identified proteins were sorted into KEGG metabolic maps (S7 Table) in order to confirm the previous COG results and give further support to our hypothesis that experimental diets affect the protein expression of the microbial community. The BD+ metaproteomes differ from the BD—counterpart basically in the pyrimidine metabolism, where the BD+ microbial community is involved in the production of carbamoyl phosphate, an intermediate in the biosynthesis of arginine and the pyrimidine nucleotides. In addition, differences were observed in the two component system (KO 02020) pathway. The bacteria induced by feeding of the mineral P-supplied diets expressed PhoP, an OmpR family regulator involved in P assimilation, whereas samples of the BD—microbiota showed a higher abundance of outer membrane proteins OmpC and OmpF, involved in the passive diffusion of small molecules across the outer membrane. This suggests an attempt of the microbial community to save and/or improve the uptake of the limited available P. A study of the potential metabolic activity of the cecal microbiome showed a significant enrichment of the ‘transporters in models’ SEED subsystem in the chickens treated with coccidiostats/growth promoters when compared to the control animals [7]. Predicted proteins of this group are involved in several biological processes such as amino acid, potassium, calcium and heavy metals transport. A closer look within KOs abundant in the MP samples underline that the MP+ microbiota was active in the *de novo* synthesis of nucleotides as suggested by the identification of dihydroorotate dehydrogenase, carbamoyl-phosphate synthase and UMP kinase. MP0 microbiota by contrast, seemed to be more active in the stress response mechanisms as supported by the identification of D-proline reductase, the enzyme involved in the production of intermediate products that will enter the lysine degradation pathway. Both MP0 and MP+ metaproteomes are involved in P assimilation, but only the microbiota of the MP+ diets was concerned in energy production to support the biosynthetic metabolism. This is supported by the exclusive detection of proteins involved in aerobic/anaerobic respiration and other enzymes (beta-galactosidase, galactokinase, galT, PTS-Aga-EIIA, tagatose 1,6-diphosphate aldolase) indicating an enhanced activity on sugars digestion oriented to energy production. Additional activity of the MP+ microbiota included amino acids biosynthesis as suggested by the identification of several enzymes such as argininosuccinate lyase, glutamate synthase, phosphoglycerate dehydrogenase, glycine hydroxymethyltransferase, tryptophan synthase, glycine acetyltransferase and threonine dehydrogenase.

The differences in the abundances of certain functional pathways depending on the diet was strongly linked to changes of the microbial community composition (S8 Table). In the cecal BD+ diets for example, *Bacteroidaceae* was among the most abundant families in many pathways, in particular the glycolysis/gluconeogenesis (KO 00010), ribosome, fructose and mannose metabolism (KO 00051), and the uniquely expressed pyruvate metabolism (KO 00620) KEGG pathway. Results of the BD—diet metaproteome showed *Eubacteriaceae* as the major family, together with other bacterial families, encoding for plant-pathogen interaction and glyoxylate and dicarboxylate metabolism (KO 00630). *Lactobacillaceae* was the principal family in the MP0 treatment encoding the ABC transporter and RNA degradation (KO 03018) as the most abundant KEGG pathways. In contrast, *Ruminococcaceae* was the main family to the most abundant pathways in the MP+ diets. Comparing results of MP0 and MP+ diets, the family *Lactobacillaceae* tends to reduce its proportion with the addition of MP in the shared pathways between MP0 and MP+ diets, the opposite was found for the *Ruminococcaceae* family.

In conclusion, this is the first study describing the metaproteome of the crop and ceca bacterial communities of broilers fed with different dietary treatments. Our results proved that changes in the bacterial protein inventory were triggered by the experimental diets. The bacterial community was focused on complex and productive functions in the case of P-available diets (BD+; MP500; MP12500), which was contrary to the overall direction towards stress response in the case of P-deficient diets (BD–; MP0). The data provide key findings for further investigations aimed to design innovative poultry husbandry strategies to reduce supplementation of mineral P in the diet and to maintain a balanced microbial activity in the GIT. Nevertheless, further studies are needed to draw a complete picture of the complex activities of all GIT sections and the changes of the microbiota due to different dietary regimen. Moreover, investigation of the mucosa-associated microbiota as well as the specific bacteria involved in the InsP_6_ turnover are needed for an overall description of the chicken´s GIT microbiota and its changes triggered by the diet.

## Supporting Information

S1 File Ethical approval document of the animal experiment. (PDF)

S2 File This file includes all supplementary figures (Figure A–Figure F, see below). **Figure A Experimental workflow.** Diagram shows a general overview of the experimental workflow of this study. Orange part of the diagram highlight the main steps of the workflow. Briefly, animal experiment consists of 1140 Ross 308 broiler chicken. Out of these, 960 animals were housed in 48 pens, 20 birds each. Pens were assigned to six different experimental diets (8 pens/ diet). For the whole microbiome analyses, 6 pens/diet were first chosen. All other animals were used for other investigations. For metaproteomic analyses, 4 animals each from 2 pens/diets were randomly selected. Collected content of crop and ceca was homogenized on a pen basis yielding two crop and cecal samples per diet (biological duplicates of crop and ceca, respectively). Obtained samples were subjected to the protocols for sample preparation for tandem mass spectrometry measurements, obtained raw files were finally subjected to bioinformatics data analysis as detailed in material and methods section. **Figure B Crop and cecal metaproteome overview.** The heatmaps show Log2 intensities of the identified proteins for crop **(panel 1)** and ceca **(panel 2)** samples. Biological duplicates of each dietary treatments are distinct with A and B letters. Identified proteins of both sections are functionally categorized into KEGG biochemical pathways. KEGG pathways abbrevaiations: Gl.: Glycolysis/gluconeogenesis; Rib.: Ribosome; Pur.: Purine metabolism; Pl.-pat.: Plant-pathogen interaction; ABC: ABC transporters; Ala, Asp, Glu: Alanine, aspartate and glutamate metabolism; Pyruv.: Pyruvate metabolism; Glyox.: Glyoxylate and dicarboxylate metabolism; PentP: Pentose phosphate pathway; Fruc.: Fructose and mannose metabolism; Starch: starch and sucrose metabolism; Amin. t-RNA: Aminoacil-t-RNA biosynthesis; Galac: Galactose metabolism; One C: One carbon pool by folate; Gly, Ser, Thr: Glycine, serine and threonine metabolism; FA: Fatty acid metabolism; Pyr.: Pyrimidine metabolism; Val, Leu, Ile: Valine, leucine and isoleucine biosynthesis; Amino & nucleotide sugar: Amino sugar and nucleotide sugar metabolism; Cys Met: Cysteine and Methionine metabolism; Ox.ph.: Oxidative phosphorylation; RNA deg.: RNA degradation; Prot ER: Protein processing in endoplasmic reticulum. **Figure C Taxonomic composition at family level of the chicken´s GIT microbiota. (A)** Crop active microbiota. **(B)** Cecal active microbiota. Grey background is assigned to non-detected families. **Figure D Functional classification of the whole metaproteomes into COG categories.** Bar´s height is calculated on the basis of the Log2 abundance intensites of the identified proteins for crop **(A, B)** and ceca **(C, D)**. Biological duplicates are shown for crop **(A)** and cecal **(C)** samples. **(B and D)** charts refer to the COG categorization of the averaged duplicates of crop and ceca respectively. COG categories abbreviations: C: Energy production and conversion; E: Amino acid transport and metabolism; F: Nucleotide transport and metabolism; G: Carbohydrate transport and metabolism; H: Coenzyme transport and metabolism; I: Lipid transport and metabolism; J: Translation, ribosomal structure and biogenesis; K: Transcription; L: Replication, recombination and repair; M: Cell wall/membrane/envelope biogenesis; N: Cell motility; O: Posttranslational modification, protein turnover, chaperones; P: Inorganic ion transport and metabolism; Q: Secondary metabolites biosynthesis, transport and catabolism; R: General function prediction only; T: Signal transduction mechanisms; U: Intracellular trafficking, secretion, and vesicular transport; V: Defense mechanisms. **Figure E Functional classification of crop proteins into COG categories.** Heat-Map is drawn on the basis of the relative percentages of the proteins of each statistically different treatment. COG categories abbreviations: G: Carbohydrate transport and metabolism; J: Translation, ribosomal structure and biogenesis; R: General function prediction only; E: Amino acid transport and metabolism; C: Energy production and conversion; O: Post-translational modification, protein turnover, chaperones; P: Inorganic ion transport and metabolism; M: Cell wall/membrane/envelope biogenesis; I: Lipid transport and metabolism; U: Intracellular trafficking, secretion, and vesicular transport; K: Transcription; H: Coenzyme transport and metabolism; S: Function unknown; V: Defense mechanisms; Q: Secondary metabolites biosynthesis, transport and catabolism. **Figure F Functional characterization of the crop and cecal metaproteome.** Heat maps show the LFQ abundances of the proteins responsible of significant dissimilarities between experimental treatments in crop **(A)** and ceca **(B)**. Proteins are categorized into KEGG biochemical pathways: Gl.: Glycolysis/gluconeogenesis; Rib.: Ribosome; Pur.: Purine metabolism; Pl.-pat.: Plant-pathogen interaction; ABC: ABC transporters; Ala, Asp, Glu: Alanine, aspartate and glutamate metabolism; Pyruv.: Pyruvate metabolism; Glyox.: Glyoxylate and dicarboxylate metabolism; PentP: Pentose phosphate pathway; Fruc.: Fructose and mannose metabolism; Starch: starch and sucrose metabolism; Amin. t-RNA: Aminoacil-t-RNA biosynthesis; Galac: Galactose metabolism; One C: One carbon pool by folate; FA: Fatty acid metabolism; Pyr.: Pyrimidine metabolism; Val, Leu, Ile: Valine, leucine and isoleucine biosynthesis; Cys Met: Cysteine and Methionine metabolism; Ox.ph.: Oxidative phosphorylation; RNA deg.: RNA degradation.
(DOCX)

S1 Table Study diet composition. The two basal diets were formulated to contain adequate levels of all nutrients according to the recommendations of the German Society for Nutritional Physiology (GfE), with the exception of P and Ca. The table below show the ingredient composition and the concentration of the analyzed nutrients of the two basal diets. Table adapted from Zeller E, Schollenberger M, Witzig M, Shastak Y, Kuhn I, Hoelzle LE and Rodehutscord M. 2015. Poult Sci 94:1018–1029. doi: 10.3382/ps/pev087.
(XLSX)

S2 Table Peptide/protein inference overview. **(A)** Table report a summary of information on the peptides/proteins identified in crop section. **(B)** Table include further insights on peptide identification and their implication in protein IDs inference.
(XLSX)

S3 Table Peptide/protein inference overview. **(A)** Table report a summary of information on the peptides/proteins identified in ceca section.
(XLSX)

S4 Table Chicken´s proteome overview in the different diet treatments. The green section show an averaged estimation of the proteins and peptides number across the experimental treatments. Yellow section details the list of all proteins identified, and their respective abundance, in each replicate (A), (B) of all treatments.
(XLSX)

S5 Table Metaproteome overview in the different diet treatments. A) The green section show an averaged estimation of the proteins and peptides number across the experimental treatments. Yellow section details the list of all proteins identified, and their respective abundance, in each replicate of all treatments. Blue section refer to the number of peptides identified per protein in every treatment. B) The table summarize the effective number of proteins and peptides identified in the repliactes of all experimental treatments.
(XLSX)

S6 Table Marker proteins. Table below list the phylogenetic marker proteins which this work refer to for the phylogenetic assessment of the microbial community in the different dietary treatments in the ceca samples.
(XLSX)

S7 Table Proteins mapping into KEGG biochemical maps. KO number of the proteins belonging to the selected pathways are mapped into KEGG biochemical maps in order to obtain detailed informations on the direction of each biochemical pathway undertaken by the micrbiota kept at differnt dietary treatments. Colors of the table specify whether a given protein is found exclusively in a GIT section: crop (red), ceca (blue) or in both (green) sections or in a specific diet: MP+ (red), MP0 (blue), both diets (green),; P+ (red), P- (blue) or both diets (green).
(XLSX)

S8 Table Bacterial families involved in the identified KEGG pathways. The table summarize the bacterial families active in the crop (panel A) and cecal (panel B) microbiota. Percentual contribution of the specimens for every KEGG biochemical pathway was calculated on the basis of the proteins counting. Only KEGG pathways with a cumulative number of proteins greater than 1% of the total were considered.
(XLSX)

## References

[1] Apajalahti J, Kettunen A, Graham H. Characteristics of the gastrointestinal microbial communities, with special reference to the chicken. World's Poultry Science Journal. 2004;60(02):223–32.

[2] Oakley BB, Lillehoj HS, Kogut MH, Kim WK, Maurer JJ, Pedroso A, et al The chicken gastrointestinal microbiome. FEMS Microbiol Lett. 2014;360(2):100–12. 10.1111/1574-6968.12608 . 25263745

[3] Videnska P, Faldynova M, Juricova H, Babak V, Sisak F, Havlickova H, et al Chicken faecal microbiota and disturbances induced by single or repeated therapy with tetracycline and streptomycin. BMC veterinary research. 2013;9:30 10.1186/1746-6148-9-30 23406343 PMC3598895

[4] Waite DW, Taylor MW. Exploring the avian gut microbiota: current trends and future directions. Front Microbiol. 2015;6:673 10.3389/fmicb.2015.00673 26191057 PMC4490257

[5] Deusch S, Tilocca B, Camarinha-Silva A, Seifert J. News in livestock research—use of Omics-technologies to study the microbiota in the gastrointestinal tract of farm animals. Comput Struct Biotechnol J. 2015;13:55–63. 10.1016/j.csbj.2014.12.005 26900430 PMC4720016

[6] Stanley D, Hughes RJ, Moore RJ. Microbiota of the chicken gastrointestinal tract: influence on health, productivity and disease. Appl Microbiol Biotechnol. 2014;98(10):4301–10. 10.1007/s00253-014-5646-2 . 24643736

[7] Danzeisen JL, Kim HB, Isaacson RE, Tu ZJ, Johnson TJ. Modulations of the chicken cecal microbiome and metagenome in response to anticoccidial and growth promoter treatment. PLoS ONE. 2011;6(11):e27949 Epub 2011/11/25. 10.1371/journal.pone.0027949 PONE-D-11-11925 [pii]. 22114729 PMC3218064

[8] Qu A, Brulc JM, Wilson MK, Law BF, Theoret JR, Joens LA, et al Comparative metagenomics reveals host specific metavirulomes and horizontal gene transfer elements in the chicken cecum microbiome. PLoS One. 2008;3(8):e2945 10.1371/journal.pone.0002945 18698407 PMC2492807

[9] Sergeant MJ, Constantinidou C, Cogan TA, Bedford MR, Penn CW, Pallen MJ. Extensive microbial and functional diversity within the chicken cecal microbiome. PLoS One. 2014;9(3):e91941 10.1371/journal.pone.0091941 24657972 PMC3962364

[10] Singh KM, Shah TM, Reddy B, Deshpande S, Rank DN, Joshi CG. Taxonomic and gene-centric metagenomics of the fecal microbiome of low and high feed conversion ratio (FCR) broilers. Journal of applied genetics. 2014;55(1):145–54. 10.1007/s13353-013-0179-4 . 24136777

[11] Tang Y, Underwood A, Gielbert A, Woodward MJ, Petrovska L. Metaproteomics analysis reveals the adaptation process for the chicken gut microbiota. Appl Environ Microbiol. 2014;80(2):478–85. 10.1128/AEM.02472-13 24212578 PMC3911106

[12] Polansky O, Sekelova Z, Faldynova M, Sebkova A, Sisak F, Rychlik I. Important Metabolic Pathways and Biological Processes Expressed by Chicken Cecal Microbiota. Applied and environmental microbiology. 2016;82(5):1569–76. Epub 2015/12/30. 10.1128/aem.03473-15 ; PubMed Central PMCID: PMCPmc4771310. 26712550 PMC4771310

[13] Konietzny U, Greiner R. PHYTIC ACID | Nutritional Impact In: Caballero B, editor. Encyclopedia of Food Sciences and Nutrition (Second Edition). Oxford: Academic Press; 2003 p. 4555–63.

[14] Zeller E, Schollenberger M, Witzig M, Shastak Y, Kuhn I, Hoelzle LE, et al Interactions between supplemented mineral phosphorus and phytase on phytate hydrolysis and inositol phosphates in the small intestine of broilers. Poult Sci. 2015;94(5):1018–29. 10.3382/ps/pev087 . 25810408

[15] Huber K, Zeller E, Rodehutscord M. Modulation of small intestinal phosphate transporter by dietary supplements of mineral phosphorus and phytase in broilers. Poult Sci. 2015;94(5):1009–17. 10.3382/ps/pev065 . 25834252

[16] Onyango EM, Asem EK, Adeola O. Dietary cholecalciferol and phosphorus influence intestinal mucosa phytase activity in broiler chicks. British poultry science. 2006;47(5):632–9. 10.1080/00071660600963651 . 17050109

[17] Ptak A, Bedford MR, Swiatkiewicz S, Zyla K, Jozefiak D. Phytase modulates ileal microbiota and enhances growth performance of the broiler chickens. PloS one. 2015;10(3):e0119770 Epub 2015/03/18. 10.1371/journal.pone.0119770 . 25781608 PMC4363628

[18] Dostal A, Fehlbaum S, Chassard C, Zimmermann MB, Lacroix C. Low iron availability in continuous in vitro colonic fermentations induces strong dysbiosis of the child gut microbial consortium and a decrease in main metabolites. FEMS microbiology ecology. 2013;83(1):161–75. 10.1111/j.1574-6941.2012.01461.x 22845175 PMC3511601

[19] Metzler-Zebeli BU, Mann E, Schmitz-Esser S, Wagner M, Ritzmann M, Zebeli Q. Changing dietary calcium-phosphorus level and cereal source selectively alters abundance of bacteria and metabolites in the upper gastrointestinal tracts of weaned pigs. Applied and environmental microbiology. 2013;79(23):7264–72. Epub 2013/09/17. 10.1128/aem.02691-13 ; PubMed Central PMCID: PMCPmc3837733. 24038702 PMC3837733

[20] Witzig M, Camarinha-Silva A, Green-Engert R, Hoelzle K, Zeller E, Seifert J, et al Spatial Variation of the Gut Microbiota in Broiler Chickens as Affected by Dietary Available Phosphorus and Assessed by T-RFLP Analysis and 454 Pyrosequencing. PLoS One. 2015;10(12):e0145588 10.1371/journal.pone.0145588 26681437 PMC4682969

[21] Abdel-Fattah WR, Chen Y, Eldakak A, Hulett FM. Bacillus subtilis phosphorylated PhoP: direct activation of the E(sigma)A- and repression of the E(sigma)E-responsive phoB-PS+V promoters during pho response. J Bacteriol. 2005;187(15):5166–78. Epub 2005/07/21. 10.1128/jb.187.15.5166-5178.2005 16030210 PMC1196004

[22] Apajalahti JH, Sarkilahti LK, Maki BR, Heikkinen JP, Nurminen PH, Holben WE. Effective recovery of bacterial DNA and percent-guanine-plus-cytosine-based analysis of community structure in the gastrointestinal tract of broiler chickens. Applied and environmental microbiology. 1998;64(10):4084–8. Epub 1998/10/06. ; PubMed Central PMCID: PMCPmc106608. 9758849 10.1128/aem.64.10.4084-4088.1998 PMC106608

[23] Neubauer G, Mann M. Mapping of phosphorylation sites of gel-isolated proteins by nanoelectrospray tandem mass spectrometry: potentials and limitations. Analytical chemistry. 1999;71(1):235–42. Epub 1999/01/28. . 9921130 10.1021/ac9804902

[24] Jehmlich N, Schmidt F, Hartwich M, von Bergen M, Richnow HH, Vogt C. Incorporation of carbon and nitrogen atoms into proteins measured by protein-based stable isotope probing (Protein-SIP). Rapid communications in mass spectrometry: RCM. 2008;22(18):2889–97. Epub 2008/08/30. 10.1002/rcm.3684 . 18727149

[25] Hansen SH, Stensballe A, Nielsen PH, Herbst FA. Metaproteomics: Evaluation of protein extraction from activated sludge. Proteomics. 2014;14(21–22):2535–9. 10.1002/pmic.201400167 . 25116144

[26] Jagtap P, Goslinga J, Kooren JA, McGowan T, Wroblewski MS, Seymour SL, et al A two-step database search method improves sensitivity in peptide sequence matches for metaproteomics and proteogenomics studies. Proteomics. 2013;13(8):1352–7. 10.1002/pmic.201200352 23412978 PMC3633484

[27] Vizcaino JA, Csordas A, Del-Toro N, Dianes JA, Griss J, Lavidas I, et al 2016 update of the PRIDE database and its related tools. Nucleic Acids Res. 2016;44(D1):D447–56. 10.1093/nar/gkv1145 26527722 PMC4702828

[28] Bray JR, Curtis JT. An Ordination of the Upland Forest Communities of Southern Wisconsin. Ecological Monographs. 1957;27(4):325–49. 10.2307/1942268

[29] Clarke KR. Non-parametric multivariate analyses of changes in community structure. Australian Journal of Ecology. 1993;18(1):117–43. 10.1111/j.1442-9993.1993.tb00438.x

[30] Wu S, Zhu Z, Fu L, Niu B, Li W. WebMGA: a customizable web server for fast metagenomic sequence analysis. BMC Genomics. 2011;12:444 10.1186/1471-2164-12-444 21899761 PMC3180703

[31] Segata N, Izard J, Waldron L, Gevers D, Miropolsky L, Garrett WS, et al Metagenomic biomarker discovery and explanation. Genome Biol. 2011;12(6):R60 10.1186/gb-2011-12-6-r60 21702898 PMC3218848

[32] Venables. Gplots: various R programming tools for plotting data. R-package. 2.7.4 ed2009.

[33] Tanca A, Palomba A, Pisanu S, Addis MF, Uzzau S. Enrichment or depletion? The impact of stool pretreatment on metaproteomic characterization of the human gut microbiota. Proteomics. 2015 Epub 2015/02/14. 10.1002/pmic.201400573 . 25677681

[34] Haange SB, Oberbach A, Schlichting N, Hugenholtz F, Smidt H, von Bergen M, et al Metaproteome analysis and molecular genetics of rat intestinal microbiota reveals section and localization resolved species distribution and enzymatic functionalities. J Proteome Res. 2012;11(11):5406–17. Epub 2012/09/29. 10.1021/pr3006364 . 23016992

[35] Deusch S, Seifert J. Catching the tip of the iceberg—evaluation of sample preparation protocols for metaproteomic studies of the rumen microbiota. Proteomics. 2015 10.1002/pmic.201400556 . 25765363

[36] Yu CS, Chen YC, Lu CH, Hwang JK. Prediction of protein subcellular localization. Proteins. 2006;64(3):643–51. Epub 2006/06/06. 10.1002/prot.21018 . 16752418

[37] Sekelja M, Rud I, Knutsen SH, Denstadli V, Westereng B, Naes T, et al Abrupt temporal fluctuations in the chicken fecal microbiota are explained by its gastrointestinal origin. Applied and environmental microbiology. 2012;78(8):2941–8. Epub 2012/02/07. 10.1128/aem.05391-11 22307311 PMC3318845

[38] Simon C, Daniel R. Metagenomic analyses: past and future trends. Applied and environmental microbiology. 2011;77(4):1153–61. Epub 2010/12/21. 10.1128/aem.02345-10 21169428 PMC3067235

[39] Venter JC, Remington K, Heidelberg JF, Halpern AL, Rusch D, Eisen JA, et al Environmental genome shotgun sequencing of the Sargasso Sea. Science (New York, NY). 2004;304(5667):66–74. Epub 2004/04/07. 10.1126/science.1093857 . 15001713

[40] Ochi K. A taxonomic study of the genus Streptomyces by analysis of ribosomal protein AT-L30. Int J Syst Bacteriol. 1995;45(3):507–14. Epub 1995/07/01. 10.1099/00207713-45-3-507 8590678

[41] Korobeinikova AV, Garber MB, Gongadze GM. Ribosomal proteins: structure, function, and evolution. Biochemistry (Mosc). 2012;77(6):562–74. Epub 2012/07/24. 10.1134/s0006297912060028 . 22817455

[42] Lecomte V, Kaakoush NO, Maloney CA, Raipuria M, Huinao KD, Mitchell HM, et al Changes in gut microbiota in rats fed a high fat diet correlate with obesity-associated metabolic parameters. PloS one. 2015;10(5):e0126931 Epub 2015/05/21. 10.1371/journal.pone.0126931 25992554 PMC4436290

[43] Hooda S, Boler BM, Serao MC, Brulc JM, Staeger MA, Boileau TW, et al 454 pyrosequencing reveals a shift in fecal microbiota of healthy adult men consuming polydextrose or soluble corn fiber. The Journal of nutrition. 2012;142(7):1259–65. Epub 2012/06/01. 10.3945/jn.112.158766 . 22649263

[44] den Besten G, van Eunen K, Groen AK, Venema K, Reijngoud D-J, Bakker BM. The role of short-chain fatty acids in the interplay between diet, gut microbiota, and host energy metabolism. Journal of Lipid Research. 2013;54(9):2325–40. 10.1194/jlr.R036012. PMC3735932. 23821742 PMC3735932

[45] Gong J, Si W, Forster RJ, Huang R, Yu H, Yin Y, et al 16S rRNA gene-based analysis of mucosa-associated bacterial community and phylogeny in the chicken gastrointestinal tracts: from crops to ceca. FEMS microbiology ecology. 2007;59(1):147–57. Epub 2007/01/20. 10.1111/j.1574-6941.2006.00193.x . 17233749

[46] Janssen PH. Identifying the dominant soil bacterial taxa in libraries of 16S rRNA and 16S rRNA genes. Applied and environmental microbiology. 2006;72(3):1719–28. Epub 2006/03/07. 10.1128/aem.72.3.1719-1728.2006 16517615 PMC1393246

[47] Zarraonaindia I, Owens SM, Weisenhorn P, West K, Hampton-Marcell J, Lax S, et al The soil microbiome influences grapevine-associated microbiota. mBio. 2015;6(2). Epub 2015/03/26. 10.1128/mBio.02527-14 25805735 PMC4453523

[48] Peris-Bondia F, Latorre A, Artacho A, Moya A, D'Auria G. The Active Human Gut Microbiota Differs from the Total Microbiota. PloS one. 2011;6(7):e22448 10.1371/journal.pone.0022448. PMC3145646. 21829462 PMC3145646

[49] Kang SS, Jeraldo PR, Kurti A, Miller MEB, Cook MD, Whitlock K, et al Diet and exercise orthogonally alter the gut microbiome and reveal independent associations with anxiety and cognition. Molecular Neurodegeneration. 2014;9:36-. 10.1186/1750-1326-9-36. PMC4168696. 25217888 PMC4168696

[50] Rooks MG, Veiga P, Wardwell-Scott LH, Tickle T, Segata N, Michaud M, et al Gut microbiome composition and function in experimental colitis during active disease and treatment-induced remission. The ISME journal. 2014;8(7):1403–17. Epub 2014/02/07. 10.1038/ismej.2014.3 24500617 PMC4069400

[51] Singh KM, Shah TM, Reddy B, Deshpande S, Rank DN, Joshi CG. Taxonomic and gene-centric metagenomics of the fecal microbiome of low and high feed conversion ratio (FCR) broilers. Journal of applied genetics. 2014;55(1):145–54. Epub 2013/10/19. 10.1007/s13353-013-0179-4 . 24136777

[52] Kostic AD, Howitt MR, Garrett WS. Exploring host—microbiota interactions in animal models and humans. Genes & Development. 2013;27(7):701–18. 10.1101/gad.212522.112. PMC3639412. 23592793 PMC3639412

[53] Eckburg PB, Bik EM, Bernstein CN, Purdom E, Dethlefsen L, Sargent M, et al Diversity of the human intestinal microbial flora. Science. 2005;308(5728):1635–8. Epub 2005/04/16. 10.1126/science.1110591 15831718 PMC1395357

