# Supplementary material for: Variations of Phosphorous Accessibility Causing Changes in Microbiome Functions in the Gastrointestinal Tract of Chickens
Source: PLoS One. 2016 Oct 19;11(10):e0164735. doi: 10.1371/journal.pone.0164735 (PMC5070839; doi:10.1371/journal.pone.0164735)
Supplement: S1 File — (PDF) [file pone.0164735.s001.pdf]

UNIVERSITÄT HOHENHEIM
TIERSCHUTZBEAUFTRAGTER
Prof. Dr. W. M. Amselgruber

Universität Hohenheim, D-70593 Stuttgart
Telefon: 0711 / 459 – 22410

Universität Hohenheim
Inst. 450
Eing.: 11. Okt. 2012
Erl.: Ro

Alen ✓
Ø Dr. W.-Fij ✓
Jagdmann ✓

Herrn
Prof. Rodehutscord
Inst. für Tierernährung -450a-

10.10.2012

Interne Versuchsanzeige

Sehr geehrter Herr Prof. Rodehutscord,

Der von Ihnen eingebrachte intern anzeigenpflichtige Versuch *„Untersuchungen wie sich die Zulage von anorganischem Phosphat, allein oder in Kombination mit Phytase, auf die Entstehung von Inositolphosphaten und die Zusammensetzung der Mikrobiota im Verdauungstrakt von Broilern auswirkt"* wird intern unter der Nummer **S 363/12 TE** geführt.
Mit Erhalt dieses Schreibens kann der Versuch begonnen werden.
Bitte verwenden sie dieses Aktenzeichen bei allen diesen Versuch betreffenden Korrespondenzen.
Bitte reichen sie die unterschriebenen Formulare (Erklärung, Stellungnahme Ausschussvorsitzender) nach

Die beiliegende Anzeige der Tötung von Wirbeltieren zu wissenschaftlichen Zwecken wird von der Zentralen Versuchstierdokumentation unter der Versuchsnummer: **T98/12 TE** erfasst.
Bitte melden Sie der Zentralen Versuchstierdokumentation über das Tiermeldeformular unter Angabe der Versuchsnummer die jeweils verbrauchten Tiere.

Mit freundlichen Grüßen

i.A. Karen Müller
