# Supplementary material for: Variations of Phosphorous Accessibility Causing Changes in Microbiome Functions in the Gastrointestinal Tract of Chickens
Source: PLoS One. 2016 Oct 19;11(10):e0164735. doi: 10.1371/journal.pone.0164735 (PMC5070839; doi:10.1371/journal.pone.0164735)
Supplement: S2 File — Figure A Experimental workflow. Diagram shows a general overview of the experimental workflow of this study. Orange part of the diagram highlight the main steps of the workflow. Briefly, animal experiment consists of 1140 Ross 308 broiler chicken. Out of these, 960 animals were housed in 48 pens, 20 birds each. Pens were assigned to six different experimental diets (8 pens/ diet). For the whole microbiome analyses, 6 pens/diet were first chosen. All other animals were used for other investigations. For metaproteomic analyses, 4 animals each from 2 pens/diets were randomly selected. Collected content of crop and ceca was homogenized on a pen basis yielding two crop and cecal samples per diet (biological duplicates of crop and ceca, respectively). Obtained samples were subjected to the protocols for sample preparation for tandem mass spectrometry measurements, obtained raw files were finally subjected to bioinformatics data analysis as detailed in material and methods section. Figure B Crop and cecal metaproteome overview. The heatmaps show Log2 intensities of the identified proteins for crop (panel 1) and ceca (panel 2) samples. Biological duplicates of each dietary treatments are distinct with A and B letters. Identified proteins of both sections are functionally categorized into KEGG biochemical pathways. KEGG pathways abbrevaiations: Gl.: Glycolysis/gluconeogenesis; Rib.: Ribosome; Pur.: Purine metabolism; Pl.-pat.: Plant-pathogen interaction; ABC: ABC transporters; Ala, Asp, Glu: Alanine, aspartate and glutamate metabolism; Pyruv.: Pyruvate metabolism; Glyox.: Glyoxylate and dicarboxylate metabolism; PentP: Pentose phosphate pathway; Fruc.: Fructose and mannose metabolism; Starch: starch and sucrose metabolism; Amin. t-RNA: Aminoacil-t-RNA biosynthesis; Galac: Galactose metabolism; One C: One carbon pool by folate; Gly, Ser, Thr: Glycine, serine and threonine metabolism; FA: Fatty acid metabolism; Pyr.: Pyrimidine metabolism; Val, Leu, Ile: Valine, leucine and is [file pone.0164735.s002.docx]

Supplementary Information

**Variations of Phosphorous Accessibility causing Changes in Microbiome Functions in the Gastrointestinal Tract of Chickens**

Bruno Tilocca^1^, Maren Witzig^1^, Markus Rodehutscord^1^, Jana Seifert^1*^

^1^ Institute of Animal Science, University of Hohenheim, Stuttgart, Germany

*** Correspondence:**

**Jana Seifert,** [**jseifert@uni-hohenheim.de**](mailto:jseifert@uni-hohenheim.de) **(JS)**

# Supplementary Figures


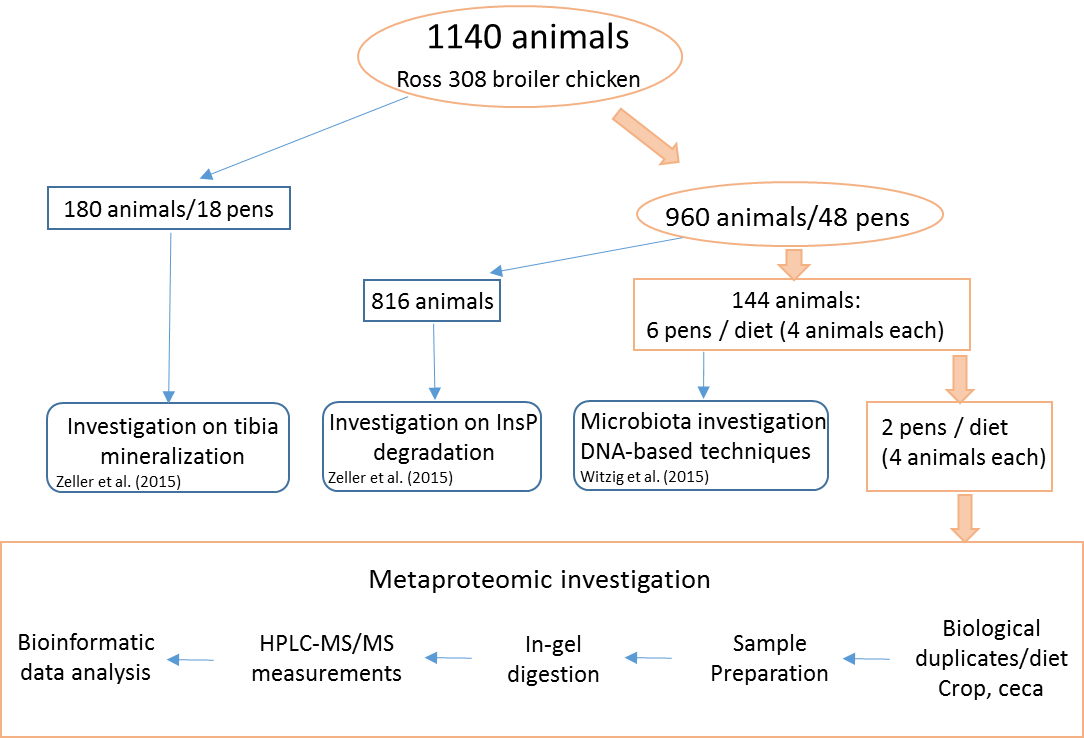


**Figure A Experimental workflow.**

Diagram shows a general overview of the experimental workflow of this study. Orange part of the diagram highlight the main steps of the workflow. Briefly, animal experiment consists of 1140 Ross 308 broiler chicken. Out of these, 960 animals were housed in 48 pens, 20 birds each. Pens were assigned to six different experimental diets (8 pens/ diet). For the whole microbiome analyses, 6 pens/diet were first chosen. All other animals were used for other investigations. For metaproteomic analyses, 4 animals each from 2 pens/diets were randomly selected. Collected content of crop and ceca was homogenized on a pen basis yielding two crop and cecal samples per diet (biological duplicates of crop and ceca, respectively). Obtained samples were subjected to the protocols for sample preparation for tandem mass spectrometry measurements, obtained raw files were finally subjected to bioinformatics data analysis as detailed in material and methods section.


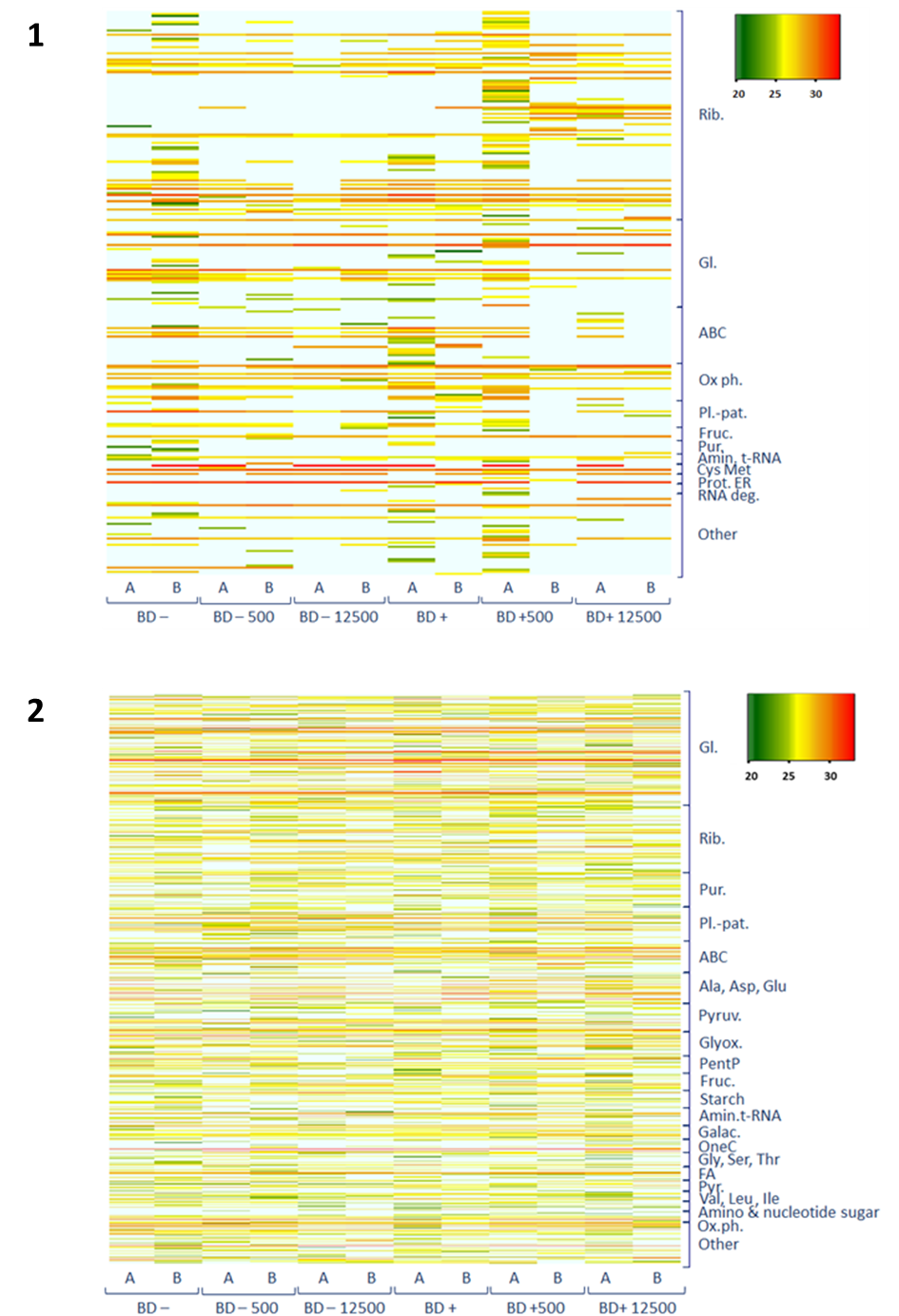


**Figure B Crop and cecal metaproteome overview.**

The heatmaps show Log2 intensities of the identified proteins for crop **(panel 1)** and ceca **(panel 2)** samples.

Biological duplicates of each dietary treatments are distinct with A and B letters.

Identified proteins of both sections are functionally categorized into KEGG biochemical pathways.

KEGG pathways abbrevaiations:

Gl. : Glycolysis/gluconeogenesis; Rib. : Ribosome; Pur. : Purine metabolism; Pl.-pat. : Plant-pathogen interaction; ABC : ABC transporters; Ala, Asp, Glu : Alanine, aspartate and glutamate metabolism; Pyruv. : Pyruvate metabolism; Glyox. : Glyoxylate and dicarboxylate metabolism; PentP : Pentose phosphate pathway; Fruc. : Fructose and mannose metabolism; Starch : starch and sucrose metabolism; Amin. t-RNA : Aminoacil-t-RNA biosynthesis; Galac : Galactose metabolism; One C : One carbon pool by folate; Gly, Ser, Thr : Glycine, serine and threonine metabolism; FA : Fatty acid metabolism; Pyr. : Pyrimidine metabolism; Val, Leu, Ile : Valine, leucine and isoleucine biosynthesis; Amino & nucleotide sugar : Amino sugar and nucleotide sugar metabolism; Cys Met : Cysteine and Methionine metabolism; Ox.ph. : Oxidative phosphorylation; RNA deg. : RNA degradation; Prot ER : Protein processing in endoplasmic reticulum.


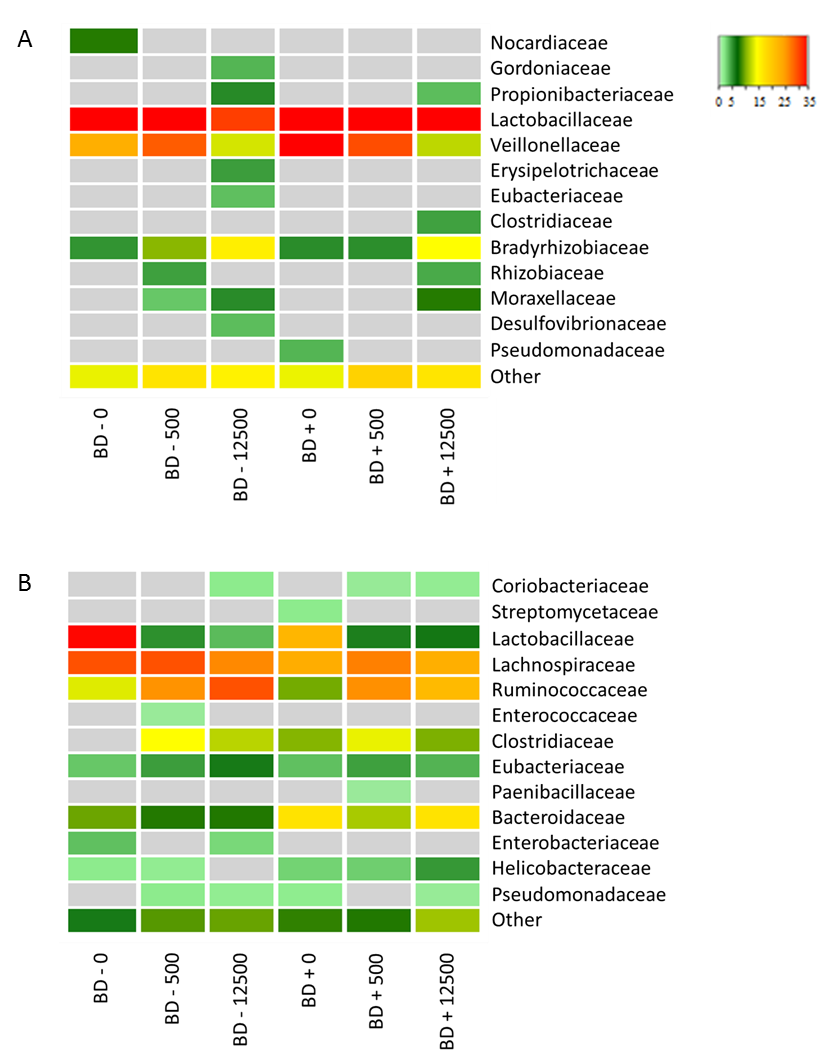


**Figure C Taxonomic composition at family level of the chicken´s GIT microbiota.**

**(A)** Crop active microbiota. **(B)** Cecal active microbiota. Grey background is assigned to non-detected families.


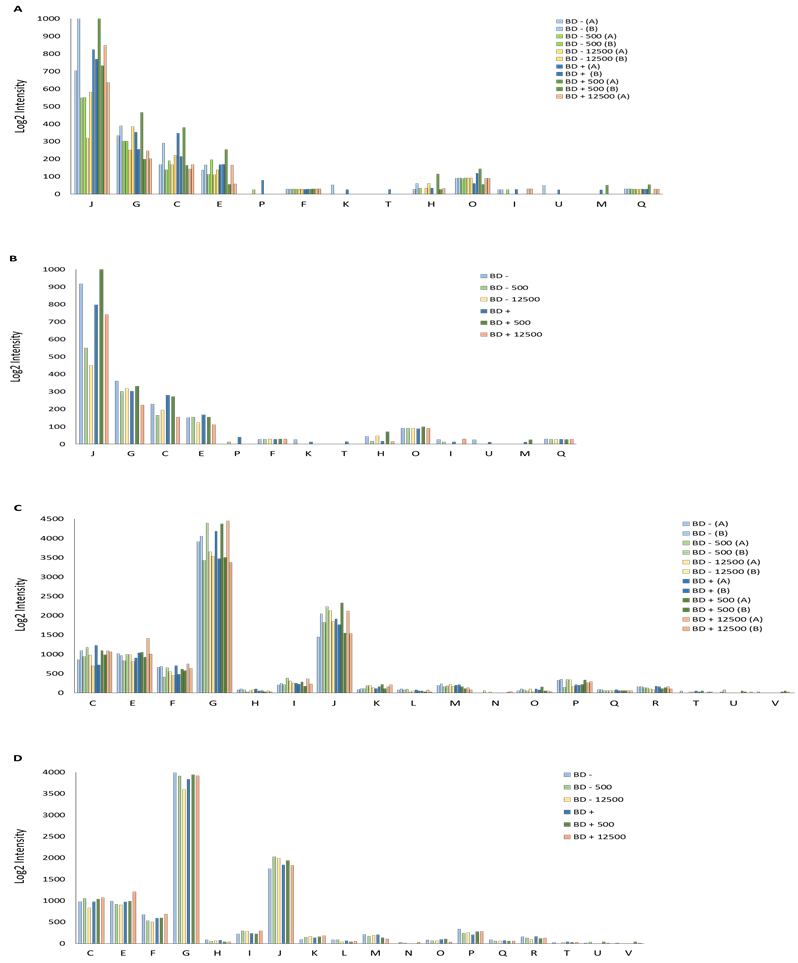


**Figure D Functional classification of the whole metaproteomes into COG categories.**

Bar´s height is calculated on the basis of the Log2 abundance intensities of the identified proteins for crop **(A, B)** and ceca **(C,D)**. Biological duplicates are shown for crop **(A)** and cecal **(C)** samples. **(B and D)** charts refer to the COG categorization of the averaged duplicates of crop and ceca respectively.

COG categories abbreviations:

C : Energy production and conversion; E : Amino acid transport and metabolism; F : Nucleotide transport and metabolism; G : Carbohydrate transport and metabolism; H : Coenzyme transport and metabolism; I : Lipid transport and metabolism; J : Translation, ribosomal structure and biogenesis; K : Transcription; L : Replication, recombination and repair; M : Cell wall/membrane/envelope biogenesis; N : Cell motility; O : Posttranslational modification, protein turnover, chaperones; P : Inorganic ion transport and metabolism; Q : Secondary metabolites biosynthesis, transport and catabolism; R : General function prediction only; T : Signal transduction mechanisms; U : Intracellular trafficking, secretion, and vesicular transport; V: Defense mechanisms.


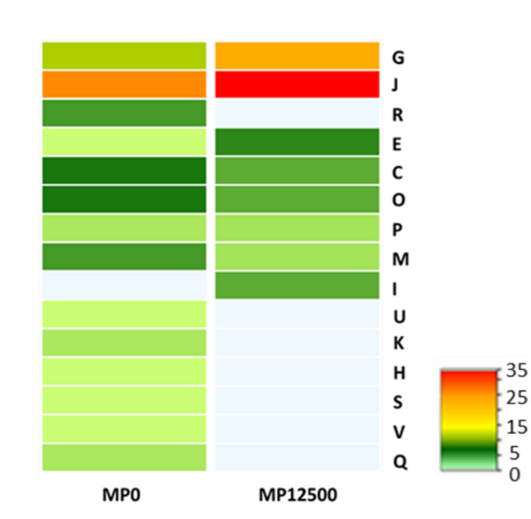


**Figure E Functional classification of crop proteins into COG categories.**

Heat-Map is drawn on the basis of the relative percentages of the proteins of each statistically different treatment.

COG categories abbreviations: G: Carbohydrate transport and metabolism; J: Translation, ribosomal structure and biogenesis; R: General function prediction only; E: Amino acid transport and metabolism; C: Energy production and conversion; O: Post-translational modification, protein turnover, chaperones; P: Inorganic ion transport and metabolism; M: Cell wall/membrane/envelope biogenesis; I: Lipid transport and metabolism; U: Intracellular trafficking, secretion, and vesicular transport; K: Transcription; H: Coenzyme transport and metabolism; S: Function unknown; V: Defense mechanisms; Q: Secondary metabolites biosynthesis, transport and catabolism.


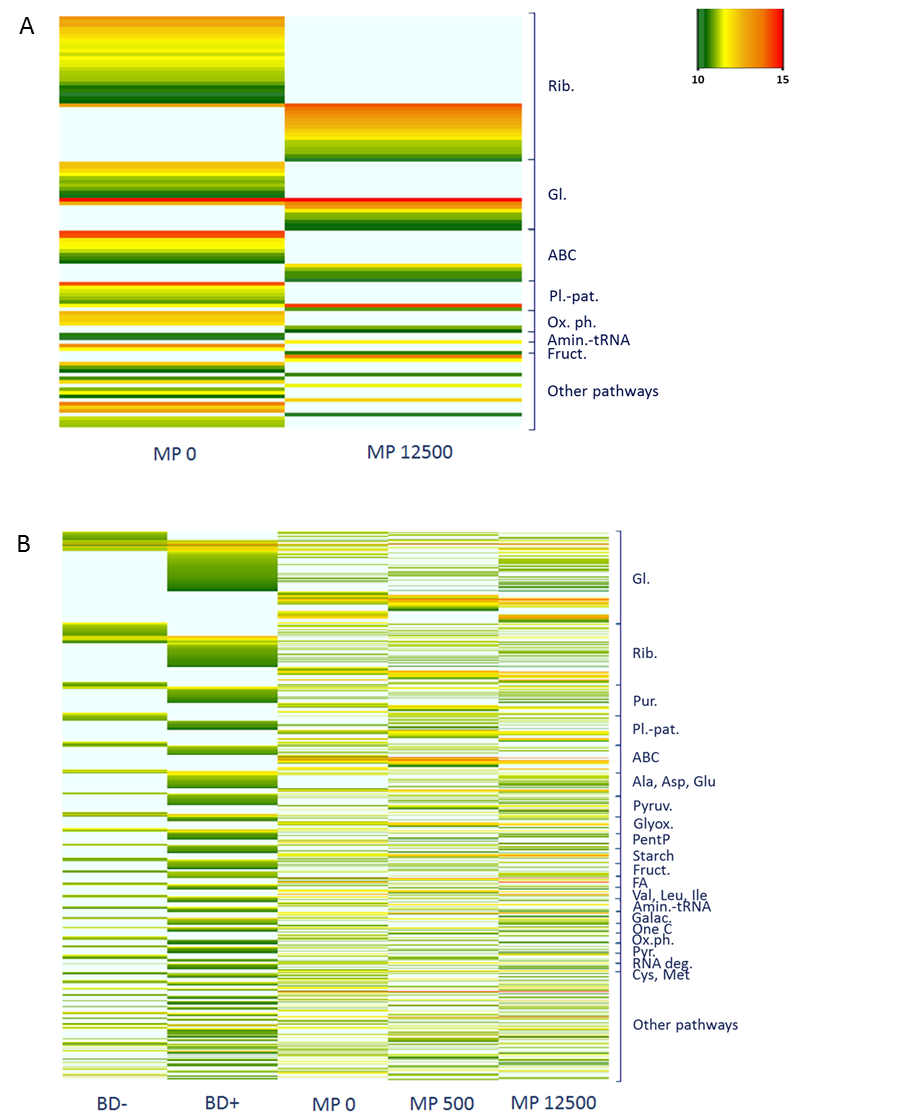


**Figure F Functional characterization of the crop and cecal metaproteome.**

Heat maps show the LFQ abundances of the proteins responsible of significant dissimilarities between experimental treatments in crop **(A)** and ceca **(B)**. Proteins are categorized into KEGG biochemical pathways: Gl.: Glycolysis/gluconeogenesis; Rib.: Ribosome; Pur.: Purine metabolism; Pl.-pat.: Plant-pathogen interaction; ABC: ABC transporters; Ala, Asp, Glu: Alanine, aspartate and glutamate metabolism; Pyruv.: Pyruvate metabolism; Glyox.: Glyoxylate and dicarboxylate metabolism; PentP: Pentose phosphate pathway; Fruc.: Fructose and mannose metabolism; Starch: starch and sucrose metabolism; Amin. t-RNA: Aminoacil-t-RNA biosynthesis; Galac: Galactose metabolism; One C: One carbon pool by folate; FA: Fatty acid metabolism; Pyr.: Pyrimidine metabolism; Val, Leu, Ile: Valine, leucine and isoleucine biosynthesis; Cys Met: Cysteine and Methionine metabolism; Ox.ph.: Oxidative phosphorylation; RNA deg.: RNA degradation.
